# Supplementary material for: How do gender disparities in entrepreneurial aspirations emerge in Pakistan? An approach to mediation and multi-group analysis
Source: PLoS One. 2021 Dec 7;16(12):e0260437. doi: 10.1371/journal.pone.0260437 (PMC8651106; doi:10.1371/journal.pone.0260437)
Supplement: S1 Questionnaire — (DOCX) [file pone.0260437.s002.docx]

| Questionnaire Gender Disparities of Entrepreneurship in Pakistan | | | | | | | | | | | | | |
| --- | --- | --- | --- | --- | --- | --- | --- | --- | --- | --- | --- | --- | --- |
| Please specify your answer by placing a tick (√) on the relevant solutions provided. Coding | | | | | | | | | | | | | |
| Q1 | Gender | | Male | | | | | | 1 | | | | |
|  |  |  | Female | | | | | | 0 | | | | |
| Q2 | Age Group | | <21 | 1 | 2 | | 3 | | 4 | | | 5 | |
|  |  |  | 21-30 | 1 | 2 | | 3 | | 4 | | | 5 | |
|  |  |  | 31-40 | 1 | 2 | | 3 | | 4 | | | 5 | |
|  |  |  | 41-50 | 1 | 2 | | 3 | | 4 | | | 5 | |
|  |  |  | >50 | 1 | 2 | | 3 | | 4 | | | 5 | |
| Q3 | Study Year | | 1st Year | 1 | 2 | | 3 | | 4 | | | 5 | |
|  |  |  | 2nd Year | 1 | 2 | | 3 | | 4 | | | 5 | |
|  |  |  | 3rd Year | 1 | 2 | | 3 | | 4 | | | 5 | |
|  |  |  | 4th Year | 1 | 2 | | 3 | | 4 | | | 5 | |
| Q4 | Level of Degree | | Under Graduate | 1 | 2 | | 3 | | 4 | | | 5 | |
|  |  |  | Master Graduate | 1 | 2 | | 3 | | 4 | | | 5 | |
|  |  |  | Doctoral Graduate | 1 | 2 | | 3 | | 4 | | | 5 | |
| SECTION -B PROBABILITY OF INDIVIDUAL EXPRESSING INTENTION IN AGRICULTURE (Qs 5-9)  **Coding** | | | | | | | | | | | | | |
| Q5 | Are you satisfied with your institute providing you with Entrepreneurial Education (intention) in Agriculture? | | | | | | | | | Yes | | 1 | |
|  |  |  |  |  |  |  |  |  |  | No | | 0 | |
| Q6 | Do you believe you have Prior Entrepreneurial Knowledge of Agriculture? | | | | | | | | | Yes | | 1 | |
|  |  |  |  |  |  |  |  |  |  | No | | 0 | |
| Q7 | Whether your Parents have Entrepreneurial Exposure to setting up new agribusiness? | | | | | | | | | Yes | | 1 | |
|  |  |  |  |  |  |  |  |  |  | No | | 0 | |
| Q8 | Do you have any Prior Entrepreneurial Exposure in agriculture? | | | | | | | | | Yes | | 1 | |
|  |  |  |  |  |  |  |  |  |  | No | | 0 | |
| Q9 | Do you have any Prior Farming Exposure to operating Agriculture very well? | | | | | | | | | Yes | | 1 | |
|  |  |  |  |  |  |  |  |  |  | No | | 0 | |
| SECTION -C FACTORS THAT INFLUENCE STUDENTS’ INTENTION TOWARDS AGRICULTURE ENTREPRENEURSHIP (Qs, 10-27) | | | | | | | | | | | | | |
| **C1 PSYCHOLOGICAL FACTORS** | | | | | | **DA** | | D | | | N | A | SA |
| (SD = Strongly Disagree D = Disagree N = Neutral A = Agree SA = Strongly Agree) | | | | | | 1 | | 2 | | | 3 | 4 | 5 |
| **Entrepreneurship Intention (EI)** | | | | | | 1 | | 2 | | | 3 | 4 | 5 |
| Q10 | EI1 | My professional goal is to become an entrepreneur in the agriculture | | | |  | |  | | |  |  |  |
| Q11 | EI2 | I am determined to create an agriculture firm in the future | | | |  | |  | | |  |  |  |
| Q12 | EI3 | I have the firm intention to start an agriculture firm someday | | | |  | |  | | |  |  |  |
| **Attitudes toward Behavior (ATB)** | | | | | | 1 | | 2 | | | 3 | 4 | 5 |
| Q13 | ATB1 | Being an agriculture entrepreneur implies more advantages than disadvantages to me | | | |  | |  | | |  |  |  |
| Q14 | ATB2 | If I had the opportunity and resources, I’d like to start a firm in the agriculture | | | |  | |  | | |  |  |  |
| Q15 | ATB3 | Among various options, I would rather be an agriculture entrepreneur | | | |  | |  | | |  |  |  |
| **Subjective norms (SN)** | | | | | | 1 | | 2 | | | 3 | 4 | 5 |
| Q16 | SN1 | I care what people who are important to me think when I decide whether to pursue a self-employed career. | | | |  | |  | | |  |  |  |
| Q17 | SN2 | I care what my closest friends think when I decide whether or not to pursue a career as agri-entrepreneurship | | | |  | |  | | |  |  |  |
| Q18 | SN3 | My parents are positively oriented toward a career as an agriculture entrepreneur logical choice for me | | | |  | |  | | |  |  |  |
| **Perceived behavioral control (PBC)** | | | | | | 1 | | 2 | | | 3 | 4 | 5 |
| Q19 | PBC1 | If I pursue a career in agriculture as self-employed, the chances of failure would be | | | |  | |  | | |  |  |  |
| Q20 | PBC2 | The number of events outside my control, which could prevent me from being self-employed in agriculture | | | |  | |  | | |  |  |  |
| Q21 | PBC3 | As self-employed in the agriculture sector, I have complete control over the situation. | | | |  | |  | | |  |  |  |
| **Entrepreneurial Education (EE)** | | | | | | 1 | | 2 | | | 3 | 4 | 5 |
| Q22 | EE1 | I tend to read books on how to set up a firm. | | | |  | |  | | |  |  |  |
| Q23 | EE2 | I prefer to try to spend time learning about starting a firm. | | | |  | |  | | |  |  |  |
| Q24 | EE3 | I usually attend seminars and conferences that focus on starting my business planning. | | | |  | |  | | |  |  |  |
| **Personality Traits (PT)** | | | | | | 1 | | 2 | | | 3 | 4 | 5 |
| Q25 | PT1 | Whether or not I am successful in life depends mainly on my ability | | | |  | |  | | |  |  |  |
| Q26 | PT2 | I can spot a good opportunity long before others can | | | |  | |  | | |  |  |  |
| Q27 | PT3 | I can take risks with my money, such as investing in stocks | | | |  | |  | | |  |  |  |
