# Supplementary material for: How do gender disparities in entrepreneurial aspirations emerge in Pakistan? An approach to mediation and multi-group analysis
Source: PLoS One. 2021 Dec 7;16(12):e0260437. doi: 10.1371/journal.pone.0260437 (PMC8651106; doi:10.1371/journal.pone.0260437)
Supplement: Attachment — Submitted filename: Recommendation.docx [file pone.0260437.s003.docx]

acThe study aims to find out the **“How do Gender disparities in entrepreneurial aspirations emerge in Pakistan? An Approach to Mediational and Multigroup Analysis”.** The topic is interested in the field. However, some considerations need to be explained for improving the quality of the manuscript and after minor explanations and corrections, the paper could be published:

1. The abstract is quite long. Try to comprehend and concise it by interdicting the reason why you envisioned doing this research along with how and what the findings are.
2. Second, the introduction section fails to deliver the content of the study in an effective way. I find it difficult to follow the introduction. The authors should re-structure the introduction and clarify what the study aims to deliver.
3. Does the paper demonstrate an adequate understanding of the relevant literature in the field and cite an appropriate range of literature sources? Clarify it.
4. Contributions and objectives of the study need to be more clear and precise in the intro and research question need to be answered and explained clearly in conclusion.
5. Tables and Figures should be revised. I recommend the authors employ a conventional way of using tables and figures in research articles. For instance, Table 1 is written as Table I. Table 2 is written as Table 2. I would suggest to add the figures in the main text if possible.
6. There are various results which are given without the indication of significance level of specific values in relevant tables.
7. Then the present results need to be compared to the extant policy actions - the conclusions section needs to be extended. There is a need to identify the theoretical as well as the empirical contributions of your research.
8. In my opinion it is not fair to give general suggestions for whole country just on the basis of a very small sample.
9. Limitations of data collection and of the proposed model are missing in the overall paper.
